# Creation of a New Frailty Scale in Primary Care: The Zulfiqar Frailty Scale (ZFS)

**DOI:** 10.3390/medicines8040019

**Published:** 2021-04-13

**Authors:** Abrar-Ahmad Zulfiqar

**Affiliations:** Internal Medicine Department, University Hospital of Strasbourg, 67000 Strasbourg, France; abzulfiqar@gmail.com

**Keywords:** frailty, elderly, primary care, Zulfiqar Frailty Scale (ZFS)

## Abstract

**Introduction:** Very few frailty scales are used by general practitioners as they are time consuming and cumbersome. We designed a new scale for the rapid detection of frailty. **Methods:** We developed a frailty screening tool for use in primary care, referred to as the Zulfiqar Frailty Scale (ZFS). This scale was tested in a general practitioner’s office for six months in Plancoët, France. Only patients over 75 years of age with Activities of Daily Living (ADL) ≥4 were included. The objective of this research was to validate the scale, evaluate its performance, and compare this screening tool with other scales such as the Fried Scale, the Gerontopole Frailty Screening Tool (GFST), the modified Short Emergency Geriatric Assessment (mSEGA) Grid A, and the Comprehensive Geriatric Assessment (CGA). **Results:** A total of 102 patients were included, with a mean age of 82.65 ± 4.79; 55 were women and 47 were men. The percentage of frail subjects was 63.7% in our scale, 67.7% in the mSEGA grid A, 75.5% in the GFST, and 60.8% for the Fried criteria. After a comprehensive geriatric assessment, frailty syndrome was found in 57 patients (55.9%). In general, both scales showed solid performance, and differences between them in the sample were minimal. As the CGA showed a prevalence of frailty of 55.9%, a similar prevalence threshold for the ZFS (i.e., 64% at the threshold ≥3 could be assessed). The completion time for our scale was less than two minutes, and staff required no training beforehand. Its sensitivity was 83.9%, and its specificity was 67.5%. Its positive predictive value was 80%, and its negative predictive value was 73%. The Pearson correlations between the geriatric scores were all strong and roughly equivalent to each other. **Conclusions:** Our frailty screening scale is simple, relevant, and rapid (taking less than two minutes).

## 1. Introduction

Aging is a slow, progressive, and uneven process that depends on the physical, psychological, genetic, and environmental variability of each person. While older people experience “successful” or “typical” aging, others will progressively lose autonomy.

Described since the 70s, frailty syndrome is a dynamic and evolving geriatric concept involving numerous dimensions of everyday life and leading to the risk of developing a loss of autonomy. It corresponds to a precarious state of equilibrium linked to the reduction of physiological reserves due to aging and is responsible for the inability to respond to physical, psychological, or social stress. Its management requires medical, social, and psychological interventions [1].

Regardless of the models used for estimating the aging of the French population, the results converge: the percentage of people aged 65 and over will increase sharply until 2040. Two causes have been well identified: The Baby Boom generation reaching this age group and the increase in life expectancy. By 2016, life expectancy will reach 85.3 years for women and 79.3 years for men, respectively. This represents an increase of 1.2 years for women and 2.2 years for men compared to 2006 [2]. According to INSEE (Institut National de la Statistique et des Études Économiques) demographic projections, one out of four inhabitants will be 65 or over in 2040, compared with 18% in 2013 [3].

In contrast, life expectancy in good health at age 65, as determined by the number of years without disability, remains stable. As of 2016, it stands at 10.5 years for women and 9.4 years for men. As a result, the rate of dependent elderly people has increased: in 2012, there were 1.2 million dependent people, and this rate will continue to increase, reaching at least two million people by 2040 [4]. Expenditures related to dependent elderly care has also increased, totaling 23.7 billion euros in 2014 versus 21.1 billion in 2011 [5].

Considering the aging of the French population, the increasing dependency of elderly persons, and the resulting health and economic consequences, frailty seems to be a major public health issue and a target for preventive medicine. 

Frailty is not a spontaneously resolving process, but it might be reversible in cases of early and targeted intervention [6]. Screening for frailty in primary care, combined with the intervention of a geriatric team if needed, has been proven to limit or even stop the development of frailty syndrome [7].

The general practitioner plays an important role in the detection and prevention of frailty in elderly individuals through early screening. However, frailty remains difficult to assess in primary care due to the multitude of conflicting definitions and the diagnostic tools available. Moreover, not all of these instruments have been validated in the context of general medicine, and their implementation is not systematically adapted for general practitioners [8]. 

Ideally, as with any screening tool, these frailty detection scores should be sensitive enough to identify as many frail individuals as possible, but also specific enough to avoid conducting a standardized geriatric test that is not very useful or efficient for those who are not frail. These scores must be validated by comparing them to benchmark tools. Finally, to be useful, a frailty detection score must be relevant for all health practitioners (whether or not they are doctors), which means we need a simple and reproducible set of criteria in order to avoid a lengthy training period beforehand. A tool with these characteristics should be easy to use.

There is no consensus regarding frailty diagnostic criteria. The prevalence of frailty depends on the tool used. In the European SHARE study, the prevalence of frailty varied from 6% to 43% depending on the eight tools used [1,2,3,4,5,6,7,8,9]. These tools were validated by international cohort studies for diagnosing frailty, but appear difficult to use in general medical practice. Due to this, we developed a tool for identifying frailty in general medicine for independent subjects over 65 years old that is intended to be quick and easy to use. It takes into account the various factors related to the risk of frailty (social, cognitive, nutritional, iatrogenic, and falls).

The objective of our study was to determine the performance of the “Zulfiqar Frailty Scale” (ZFS) tool to detect frailty (as defined by Fried’s criteria) in this ambulatory population and to compare it to other scales such as the Gerontopole Frailty Screening Tool (GFST), the modified Short Emergency Geriatric Assessment (mSEGA) Grid A, and the comprehensive geriatric assessment (CGA). Its secondary objectives were to evaluate the feasibility and acceptability of our “ZFS” tool in the setting of a primary care consultation and to evaluate the prevalence of each element of the “ZFS” composite tool in a population of older subjects seeing general practitioners.

## 2. Material and Methods

### 2.1. Study Type

A prospective 6-month study (between 1 November 2018 and 30 April 2019) was conducted by general practitioners at one primary care clinic in Brittany, France. The physicians were previously trained on the use of frailty scales such as the Fried Scale as well as on the main geriatric scales including the Activities of Daily Living (ADL), the Instrumental ADL (IADL), the Monopodal Support Test, the Mini Mental State Exam (MMSE), and nutritional assessments.

### 2.2. Frailty Screening with the “Zulfiqar Frailty Scale” (ZFS) Tool

The score was calculated by way of six indicators that measured the main functions of an elderly person [10,11,12,13] in terms of their geriatric relevance as defined by the scientific literature. A point was assigned for each positive indicator (maximum score = 6) (see Table 1). Two indicators dealt with the social aspect of aging.

- Nutritional status: weight loss of 5% or more during the previous six months;
- Physical capabilities, balance/falls: one-legged stance test. Quick and easy to perform, considered abnormal if the elderly patient could not stand on one leg for at least 5 s [10];
- Social isolation: Does the patient live alone?
- Limitations in daily living activities: Does the patient receive home care?
- Cognitive functions: Does the patient complain of memory loss? Cognitive functions were considered impaired in the event of an obvious cognitive disorder brought to light by the patient, their family or friends or a medical record; and
- Polymedicine: Does the person have prescriptions for more than 5 therapeutic classes on his/her prescription history for at least 6 months?

### 2.3. Study Population

- Inclusion criteria
  - Patients aged 75 or older;
  - Patients living at home;
  - Patients autonomous with an ADL (Activities of Daily Living) score of 4 or more (ADL >= 4/6);
  - Away from any acute pathology (infectious pathologies, falls, fractures, etc.).
- Exclusion criteria
  - Patients younger than 75 years old;
  - Patients living in nursing homes;
  - Patients with an ADL < 4/6;
  - Patients who did not provide their written consent during the introductory phase of the study;
  - Patients with recent acute illness.
- Information and Consent

Patient information was placed in the waiting rooms of general practitioners and/or consultation offices. It specified the name and type of work, the objective of the work and its context. It also specified that patients could refuse to participate in the study without affecting the quality of their care.

### 2.4. Study Parameters

#### 2.4.1. Population Characteristics

The following data were collected:

- Gender;
- Age;
- Contact person: relationship and contact information;
- ADL and IADL scores;
- Medical and surgical history;
- Charlson comorbidity score: The Charlson Comorbidity Index predicts the ten-year mortality for a patient who may have a range of comorbid conditions. The Charlson Comorbidity Index (CCI) is a commonly used scale for assessing morbidity; and
- Height, weight, and BMI (body mass index).

#### 2.4.2. Measuring Frailty with Fried’s Criteria, the GFST Tool, and the mSEGA Grid A

Fried’s scale [10] defines frailty on the basis of five criteria: fatigue, involuntary weight loss, reduced physical activity, slower walking speed, and decreased muscle strength. One point is assigned for each criterion, with patients considered “robust” or “non-frail” when none of the criteria are met; “pre-frail” when 1 or 2 of the criteria are met; and “frail” when 3 or more of the criteria are met.

The Gerontopole Frailty Screening Tool (GFST) consists of two parts: a questionnaire conducted first, and the clinician’s assessment of frailty [11,12].

The SEGA (Short Emergency Geriatric Assessment) scale was created by a Belgian team [13]. This frailty scale was initially developed for elderly subjects admitted to the emergency department [13] before being modified and validated for use with community-dwelling subjects [14,15]. The mSEGA comprises Sheet A and evaluates frailty per se on a 13-item scale, which includes: Medications/Mood/Perception of health/Falls in the previous 6 months/Nutrition/Associated diseases/Mobility/Continence/Cognitive function/Age/Place of living/IADL/Meals. Each item is graded either 0 (most favorable state) or 1 or 2 (least favorable state), thus making it possible to classify subjects into three groups: not very frail (score ≤ 8), frail (8 < score ≤ 11), and very frail (score > 11).

#### 2.4.3. Feasibility

The feasibility of the “ZFS” tool for general medicine was determined by the amount of time that was required to screen for frailty. For the sake of fairness, one of two methods was chosen at random to evaluate patients.

#### 2.4.4. Patient Acceptability

Acceptability is a set of conditions that make this test acceptable to patients. It was measured using a visual analog, non-graduated scale that was appropriate for the question being asked.

After using our (“ZFS”) tool, patients were asked to rate the acceptability of the scale between “completely unacceptable” and “completely acceptable.” The instruction given was “Place the cursor between these two options based on your opinion”.

The response was then weighted according to the same principle as the visual analog scale (VAS) for pain:

- Zero corresponded to “completely unacceptable”.
- Ten corresponded to “completely acceptable”.

#### 2.4.5. Comprehensive Geriatric Assessment (CGA)

A comprehensive geriatric assessment (CGA) was conducted for all patients by a geriatric multidisciplinary team during the geriatric consultation, and for each patient, frailty syndrome was searched for, independent of our primary care consultation. 

#### 2.4.6. Statistics

Data were collected with no identifying information on an Excel spreadsheet and analyzed using XL-Stat software. A descriptive analysis of the results was first conducted. Quantitative variables were expressed as mean ± standard deviation and qualitative variables as absolute and relative numbers (percentages). Subsequently, a comparison of our tool was conducted against other frailty scales, namely the Fried Scale, the Gerontopole Frailty Screening Tool (GFST) scale, the modified SEGA (mSEGA) grid A, and the comprehensive geriatric assessment (CGA). The receiver operating curve (ROC) was constructed using GraphPad-Prism software. This software allows for the evaluation of the performance of a diagnostic test and determines the optimal threshold values. We calculated the Pearson correlation coefficients “r” between each frailty score. These coefficients were grouped into correlation matrices. All analyses were performed with R 4.0.2 software with an alpha risk set at 5%.

### 2.5. Administrative Requirements

All patients who participated in our study were required to sign a consent form.

The research protocol was reviewed and approved by the National Commission of Information and Freedom and by the Internal Department Ethics Committee (No. 20-10-18), registration number RCB: 2017-A02563-50. 

## 3. Results

### 3.1. Characteristics of the Study Population

During our study, we offered the screening to 102 patients.

A total of 102 patients were included with a mean age of 82 years. There were 55 women (53.8%) and 47 men (see Table 2).

### 3.2. Frailty Scales

The evaluation of frailty in the study population using other scales is shown in Table 3, Table 4, Table 5 and Table 6.

### 3.3. Use of the ZFS Scale

In scoring the ZFS scale, each positive response was given a score of one point, for a maximum of six points per patient evaluation. The average score for our scale was 3.32 ± 1.55. The item “Weight loss ≥ 5% in 6 months” was found in 17 patients (16.7%); a positive response for “Fall risk according to monopodal support test” was found in 69 patients (67.6%); 40 patients responded positively (39.2%) to feeling “Lonely”; the presence of home assistance was noted in 46 subjects (45.1%); “Complaints of memory problems” was found in 67 subjects (65.7%); and 74 patients (72.5%) were positive with respect to “≥5 therapeutic classes”. An elderly patient was considered fragile when the ZFS score was ≥3. As a result, 76 subjects (74.5%) were classified as frail. The average completion time for the analysis was 109.62 s ± 9.24.

The distribution of each category is shown in Table 7, and we studied the internal consistency of our questionnaire (see Table 8 and Table 9).

### 3.4. Comprehensive Geriatric Assessment (CGA)

After a comprehensive geriatric assessment, frailty syndrome was found in 57 patients (55.9%).

### 3.5. Concordance Study

Concordance with the comprehensive geriatric assessment (CGA) is shown in Table 10.

**Figure 1:**
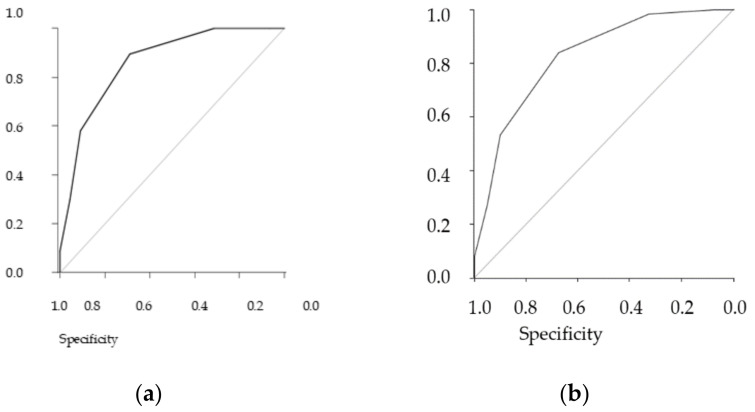
Receiver operating curve (ROC). (**a**) ZFS predictive ROC curve for CGA, AUC = 0.86, (**b**) ZFS predictive ROC curve for Fried ≥ 3, AUC = 0.83.

The table shows the results stratified by three different definitions of frailty, the performance of the GFST, and the ZFS. The gold standard is the comprehensive geriatric assessment (CGA), and we added the Fried scale as an additional reference standard (see Figure 1 (Figure 1a,b).

In general, both scales showed solid performance, and differences between them in the sample were minimal. As the CGA showed a prevalence of frailty of 55.9%, a similar prevalence threshold for the ZFS (i.e., 64% at the threshold ≥3) could be assessed (see Table 11 and Table 12 for analyses of concordance).

Sensibility was 89.5% (CI95: 78.5% to 96%), while specificity was 68.9% (CI95: 53.4% to 81.8%). The positive predictive value was 78.5% (CI95: 66.5 % to 87.7%), and the negative predictive value was 83.8% (CI95: 68% to 93.8%). 

Sensibility was 83.9% (CI95% (confidence interval): 72.3 to 92%), while specificity was 67.5% (CI95%: 50.9 to 81.4%). The positive predictive value was 80% (CI95%: 68.2 to 88.9%), and the negative predictive value was 73% (CI95%: 55.9 to 86.2%).

Whether comparing the concordance of the ZFS with the Fried or CGA scores, the results were the same.

The Pearson correlations between the geriatric scores are shown in Table 13.

### 3.6. Feasibility of the “ZFS” Tool in General Medicine

On average, it took 109.62 s ± 9.24.

Our tool was very well-received by patients, with an acceptability rating of 9.8/10 using the visual analog scale. 

## 4. Discussion

General practitioners are usually best suited to screen for frailty due to their frequent contact with elderly patients and the influence they can have on patients’ futures through their coordination of care with other health, medical, paramedical, and social service professionals. Fried’s criteria are the worldwide standard for frailty screening among elderly patients, particularly during comprehensive geriatric assessments conducted by geriatricians. However, the use of a dynamometer makes it difficult to apply these frailty screening criteria in the context of primary care. The Fried scale is widely known [10], but its inclusion in measurements is not routinely used for patient assessment. Additionally, there are no psychosocial components in the Fried scale. 

The Gerontopole Frailty Screening Tool (GFST) consists of two parts: a questionnaire conducted first, and the clinician’s assessment of frailty [11,12]. A limitation of this scale is that it does not provide specific guidance for clinicians regarding the identification of frailty. Moreover, most of the items are subjective. 

The seven-item Program of Research to Integrate the Services for the Maintenance of Autonomy (PRISMA-7) scale contains seven simple self-reported components. A total score ≥3 is considered frail [16]. It has a high level of accuracy in identifying frailty in community-dwelling older people [17], but has a tendency to over-screen for frailty [18].

The Frailty Index (FI) of Cumulative Deficits (FI-CD) was proposed by Rockwood and Mitninski. It has been well validated and has a higher predictive ability of adverse clinical events than other frailty measurements in both hospital and community settings [19,20], but it has some limitations and is time consuming. There is also a Frailty Index derived from the comprehensive geriatric assessment (CGA). It is used as a clinical standard for frailty assessment and has been found to be highly correlated with the FI-CD [21]. It is also time consuming. 

The proportion of frail individuals in our sample was similar to that obtained through the Fried and SEGA grid A scale: 63.7% in our scale, 67.7% in the SEGA grid A, 75.5% in the GFST, and 60.8% for Fried’s criteria (if at least three criteria were present). The ZFS was easy to implement and showed an appropriate level of sensitivity and negative predictive value in our study group. The tool detects frailty if at least three criteria are met. The purpose of this tool is to identify frailty in outpatients and to refer them to a geriatric team for a standardized geriatric evaluation so that they can establish a personalized plan of care (PPC). In the identification of frailty in outpatients, our scale was no less discriminating than the Fried scale. Other scales used in geriatric evaluation such as the G8 [22] have been shown to have a sensitivity similar to ours (87% for G8).

The ZFS has several advantages. It does not require previous training on the part of caregivers, and it is not time consuming, an advantage for medical consultation in elderly people. The time estimated for consultation in general medicine in France is approximately 15 min [23]. With our scale, frailty screening can be assessed in 2 min, which is similar to the GFST scale. The time is longer for the Fried scale and for the SEGA grid A (from 6 to 10 min) [6], making them more difficult for general practitioners to apply during consultation. A practical tool should require a minimum of time to complete, similar to ours. In contrast to the Fried scale, the ZFS does not require additional equipment such as a dynamometer for the determination of the isometric contraction. This is a real advantage in the context of wide-scale screening. Another advantage of the ZFS compared to the GFST scale is its higher level of objectivity for its selected criteria. Indeed, the GFST scale contains questions that are more subjective, while our tool is more objective and easy to assess. It is a quick tool that requires no prior training and no specific equipment or facilities, making it a feasible screening tool suitable for general practice. Our tool has the advantage of requiring no equipment whatsoever, making it perfectly suitable for primary care. It can be used by general practitioners, in addition to other health professionals such as nurses, physiotherapists, and occupational therapists.

### Limitations

Our study sample was small. To be validated for the purpose of studying its reproducibility, our tool will need to be tested in multiple primary care clinics, in both urban and rural areas, and across a larger sample with many types of practitioners (doctors, nurses, physical therapists, occupational therapists, etc.). The prediction of pathological events (falls, hospitalization, and morbidity-mortality) was not studied in this research. This will begin in March 2021 for an 18-month period. The cognitive question remains difficult to understand with a rapid detection score such as ours. The question “Does the person complain of memory problems?” is still subjective and requires, as we have done, a response confirmed by the patient’s family.

## 5. Conclusions

The objective of our scale is to provide the ability of conducting a rapid screening for frailty syndrome on an outpatient basis, making it possible to refer the pre-frail or frail patient to a gerontological team for the purpose of a thorough gerontological evaluation. The idea is to offer general practitioners a simple and straightforward tool for rapid screening during routine medical consultations.

Our scale must be tested further in other general practices by recruiting a wider range of participants. Eventually, the reproducibility and ability of the scale to predict potentially dangerous situations must be developed and tested on elderly patients, which will take place in the upcoming weeks and months.

## Figures and Tables

**Table 1:** Zulfiqar Frailty Scale (ZFS).

Is there a weight loss greater than or equal to 5% in 6 months?	Yes No
Monopod support test <5 s?	Yes No
Does the person live alone?	Yes No
Does the patient receive home care?	Yes No
Does the person complain of memory loss?	Yes No
Does the person have prescriptions for more than 5 therapeutic classes on his/her prescription history for at least 6 months?	Yes No

For scores of 3 or more, the elderly patient was considered by our scale to be “frail.” For scores of 1 or 2, the patient was considered “pre-frail.” For a score of 0, the patient was considered “non-frail” or “robust”.

**Table 2:** Characteristics of the study population (n = 102).

Number of Usual Drugs, Mean (SD)	6.37 (2.78)
Fall(s) in the last 6 months, n (%)	27 (26.5%)
Monopodal support test <5 s, n (%)	69 (67.6%)
Living alone, n (%)	40 (39.2%)
GDS.4, mean (SD)	1.45 (1.26)
GDS 0/4, n (%)	30 (29.4%)
GDS 1/4, n (%)	28 (27.5%)
GDS 2/4, n (%)	18 (17.6%)
GDS 3/4, n (%)	20 (19.6%)
GDS 4/4, n (%)	6 (5.9%)
MMS/30, mean (SD)	25.65 (3.9)
MNA/30, mean (SD)	12.06 (2.06)
MMS < 30, n (%)	25 (24.5%)
Charlson score, mean (SD)	3.08 (1.87)
Hospitalization in the last 6 months, n (%)	27 (26.5%)
ADL * (/6), mean (SD)	5.39 (0.73)
IADL * (/8), mean (SD)	5.52 (2.57)
BMI *, mean (SD)	27.24 (4.98)
Average number of medical histories (SD)	2.7 (1.3)
Medical History	
Cardiovascular	78 (76.5%)
Pulmonary	19 (18.6%)
Renal	18 (17.6%)
Gastrointestinal	40 (39.2%)
Endocrine	40 (39.2%)
Neurological	14 (13.7%)
Psychiatric	22 (21.6%)
Osteoarticular	50 (49%)
Oncologic	13 (12.7%)

* BMI: body mass index; * ADL: Activity Daily Living; * IADL: Instrumental Activity Daily Living; MNA: Mini Nutritional Assessment; GDS: Geriatric Depression Scale; MMSE: Mini Mental State Examination.

**Table 3:** Fried scale results in current population (n = 102).

Fried Score, Overall	Mean ± SD	2.64 ± 1.15
0	n (%)	3 (2.9%)
1 to 2	n (%)	37 (36.3%)
≥3	n (%)	62 (60.8%)

**Table 4:** Fried scale per item.

Item	n (%)
Weight loss	17 (16.7%)
Exhaustion	79 (77.5%)
Slowness	66 (64.7%)
Weakness	19 (18.6%)
Low level of physical activity	90 (88.2%)

**Table 5:** Frailty according to Gerontopole Frailty Screening Tool (GFST) scale.

Item	n (%)
Does the patient live alone?	41 (40.2%)
Has the patient involuntarily lost weight in the last 3 months?	18 (17.6%)
Has the patient experienced more fatigue in the last 3 months?	75 (73.5%)
Has the patient experienced increased mobility issues in the last 3 months?	46 (45.1%)
Has the patient complained of memory problems?	68 (66.7%)
Does the patient have a slow gait speed (i.e., >4 s to walk 4 m)?	52 (51%)
Do you think your patient is frail?	77 (75.5%)
Time GFST (seconds)	107.83 ± 9.44

**Table 6:** Frailty according to modified Short Emergency Geriatric Assessment (SEGA) grid A (n = 102).

Modified SEGA Grid A	Mean (SD)	10.26 (3.82)
0 to 8	n (%)	33 (32.4%)
9 to 11 (frail)	n (%)	31 (30.4%)
≥12 (very frail)	n (%)	38 (37.3%)

**Table 7:** Zulfiqar Frailty Scale (ZFS) score distribution (n = 102).

ZFS	Number of Subjects
0	3 (2.9%)
1	11 (10.8%)
2	23 (22.5%)
3	28 (27.5%)
4	18 (17.6%)
5	14 (13.7%)
6	5 (4.9%)

ZFS ≥ 3/6: 63.7%.

**Table 8:** Internal consistency of the questionnaire.

Is There a Weight Loss Greater Than or Equal to 5% in 6 Months?	17 (16. 7%)
Monopod support test <5 s?	69 (67.6%)
Does the person live alone?	40 (39.2%)
Are there home caregivers?	46 (45.1%)
Does the person complain of memory problems?	67 (65.7%)
Does the person have prescriptions for more than 5 therapeutic classes on his/her prescription history for at least 6 months?	74 (72.5%)

All items were informative.

**Table 9:** Item correlation matrix.

	Weight Loss	Monopod Support	Living Alone	Home Caregivers	Memory Problems	≥5 Therapeutic Classes
Weight loss	1	0.08	0.13	0.07	0.21	−0.02
Monopod support	0.08	1	0.26	0.21	0.16	0.14
Living alone	0.13	0.26	1	0.24	0.07	0.13
Home caregivers	0.07	0.21	0.24	1	0.03	0.12
Memory problems	0.21	0.16	0.07	0.03	1	0.06
≥5 therapeutic classes	−0.02	0.14	0.13	0.12	0.06	1

The following matrix presents the Phi coefficients (equivalent to Pearson correlation coefficients) between items.

**Table 10:** Concordance with the comprehensive geriatric assessment (CGA).

	ZFS Frail	ZFS Not Frail	GFST Frail	GFST Not Frail	Area under the ROC (CI95%), ZFS	Area under the ROC (CI95%), GFST
CGA	3.86 ± 1.14	2.07 ± 1.16	3.84 ± 1.11	1.8 ± 1.27	0.86 [0.79; 0.93]	0.88 [0.81; 0.94]
Fried ≥ 3	3.71 ± 1.22	2.08 ± 1.21	3.82 ± 1.09	1.57 ± 1.13	0.83 [0.74; 0.91]	0.91 [0.86; 0.97]
SEGA ≥ 12	4.29 ± 1.04	2.34 ± 1.14	4.24 ± 1.05	2.17 ± 1.28	0.89 [0.83; 0.94]	0.88 [0.82; 0.94]

**Table 11:** Contingency study ZFS/CGA.

	CGA Negative	CGA Positive
ZFS ≤ 2	31	6
ZFS ≥ 3	14	51

**Table 12:** Contingency study ZFS/FRIED.

	FRIED ≤ 2	FRIED ≥ 3
ZFS ≤ 2	27	10
ZFS ≥ 3	13	52

**Table 13:** Frailty geriatric score correlation.

Pearson Correlation	GFST	ZFS	SEGA	Fried
GFST	1	0.83	0.78	0.79
ZFS	0.83	1	0.77	0.64
SEGA	0.78	0.77	1	0.78
FRIED	0.79	0.64	0.78	1

The correlations were all strong and roughly equivalent to each other.

## Data Availability

The datasets used and/or analyzed during the current study are available from the corresponding author on reasonable request.
